# Targeted metabolomics reveals plasma short-chain fatty acids are associated with metabolic dysfunction-associated steatotic liver disease

**DOI:** 10.1186/s12876-024-03129-7

**Published:** 2024-01-23

**Authors:** Mira Thing, Mikkel Parsberg Werge, Nina Kimer, Liv Eline Hetland, Elias Badal Rashu, Puria Nabilou, Anders Ellekaer Junker, Elisabeth Douglas Galsgaard, Flemming Bendtsen, Johnny Laupsa-Borge, Adrian McCann, Lise Lotte Gluud

**Affiliations:** 1 Gastro Unit, Copenhagen University Hospital Hvidovre, Kettegard Alle 30, Hvidovre, 2650 Denmark; 2 Research & Early Development, Novo Nordisk A/S, Maaloev, 2760 Denmark; 3 Bevital AS, Bergen, Norway; 4 Department of Clinical Medicine, Faculty of Health and Medical Sciences, University of Copenhagen, København, Denmark

**Keywords:** Non-alcoholic fatty liver disease, Non-alcoholic steatohepatitis, Cirrhosis, Metabolome, Microbiome, Targeted metabolomics, Propionate, Acetate, Butyrate, Circulating SCFA

## Abstract

**Background:**

Alterations in the production of short-chain fatty acids (SCFAs) may reflect disturbances in the gut microbiota and have been linked to metabolic dysfunction-associated steatotic liver disease (MASLD). We assessed plasma SCFAs in patients with MASLD and healthy controls.

**Methods:**

Fasting venous blood samples were collected and eight SCFAs were measured using gas chromatography-tandem mass spectrometry (GC-MS/MS). Relative between-group differences in circulating SCFA concentrations were estimated by linear regression, and the relation between SCFA concentrations, MASLD, and fibrosis severity was investigated using logistic regression.

**Results:**

The study includes 100 patients with MASLD (51% with mild/no fibrosis and 49% with significant fibrosis) and 50 healthy controls. Compared with healthy controls, MASLD patients had higher plasma concentrations of propionate (21.8%, 95% CI 3.33 to 43.6, *p* = 0.02), formate (21.9%, 95% CI 6.99 to 38.9, *p* = 0.003), valerate (35.7%, 95% CI 4.53 to 76.2, *p* = 0.02), and α-methylbutyrate (16.2%, 95% CI 3.66 to 30.3, *p* = 0.01) but lower plasma acetate concentrations (− 30.0%, 95% CI − 40.4 to − 17.9, *p* < 0.001). Among patients with MASLD, significant fibrosis was positively associated with propionate (*p* = 0.02), butyrate (*p* = 0.03), valerate (*p* = 0.03), and α-methylbutyrate (*p* = 0.02). Six of eight SCFAs were significantly increased in F4 fibrosis.

**Conclusions:**

In the present study, SCFAs were associated with MASLD and fibrosis severity, but further research is needed to elucidate the potential mechanisms underlying our observations and to assess the possible benefit of therapies modulating gut microbiota.

**Supplementary Information:**

The online version contains supplementary material available at 10.1186/s12876-024-03129-7.

## Background

Metabolic dysfunction-associated steatotic liver disease (MASLD) and its progression to steatohepatitis and cirrhosis has previously been linked to the gut microbiome through various mechanisms. This association may reflect gut dysbiosis and systemic effects of gut microbiota-derived metabolites, including short-chain fatty acids (SCFAs) [1]. SCFAs are bioactive metabolites produced by bacterial fermentation of non-digestible carbohydrates and proteins in the colon [2]. SCFAs are important for gut barrier integrity and immune function, especially butyrate, which is also the primary energy source for gut epithelial cells [2–4]. In general, SCFAs play important roles in immune responses, including regulation of both the innate and adaptive immune system [5]. The mechanisms linking SCFAs and MASLD may involve alterations in glucose homeostasis, lipid metabolism, and inflammatory and immune responses [2, 6]. 

SCFAs are utilized in the colon, excreted in stool, or absorbed into the bloodstream via the portal vein [7, 8]. Beyond the gut, systemic effects of circulating SCFAs are an increasingly active area of research. Though only a small amount of gut-derived SCFAs are found to be systemically available, a significant uptake of portal propionate and butyrate by the liver has previously been found [7, 8]. In hepatic cells, propionate can be used for gluconeogenesis and acetate for *de novo* lipogenesis [2, 7, 9]. Previous studies found that fecal SCFA levels were increased in individuals with obesity and MASLD [6, 10, 11]. However, the evidence linking circulating concentrations of SCFAs to MASLD and other metabolic diseases remains unclear [12–16]. Some studies found no clear differences between controls and patients with MASLD. Other studies found lower SCFAs levels in MASLD cirrhosis or conversely observed higher levels in patients with hepatocellular carcinoma and cirrhosis related to MASLD [13, 16–18]. The evidence is therefore conflicting. The discrepancies may reflect differences in study design, such as the procedures used for selecting controls and MASLD patients, as well as the severity of the underlying MASLD. We therefore chose to investigate the association between plasma SCFAs and MASLD presenting the full range of the disease with healthy volunteers as control group. The included patients ranged from no fibrosis (F0)/mild fibrosis (F1) to severe fibrosis (F4) corresponding to cirrhosis. Accordingly, our study provides important new information about the role of SCFA in MASLD based on an evaluation of the disease at different stages of progression.

## Methods

### Study design and participants

Patients and healthy controls were included in a prospective cohort study evaluating clinical predictors and biomarkers in MASLD. The primary objectives in this study were to explore associations between plasma concentrations of individual SCFAs and the odds of having MASLD. For participants with MASLD, we evaluated the link between SCFAs and the probability of having significant fibrosis defined as F2–F4. Secondary outcomes were to estimate relative group differences in SCFA levels between MASLD patients and healthy controls, and to explore associations between SCFA concentrations and the odds of having severe steatosis, lobular inflammation, or ballooning, as well as individual fibrosis stages F0–F4.

We included 100 patients with clinically and biopsy-proven MASLD and fibrosis stage F0–F4 recruited from the outpatient clinic at the Gastro Unit Copenhagen University Hospital Hvidovre, Denmark, as well as 50 healthy volunteers recruited via advertisement. Patients and controls were matched to balance the distributions of age and sex across groups. The study was approved by The Regional Committee on Health Research Ethics (H-17,029,039) and performed in accordance with the Declaration of Helsinki [19]. Informed consent was obtained from all participants. The study was observational, and no specific dietary restrictions were used. All participants had a low alcohol intake (< 7 units/week for females and < 14 units/week for males) and none had viral hepatitis, autoimmune liver diseases, drug-induced liver disease, or other liver diseases. Patients with MASLD underwent a clinical, biochemical, and histological assessment. The histological diagnosis was made by two experienced pathologists based on the features: steatosis (0–3), lobular inflammation (0–2), hepatocellular ballooning (0–2), and fibrosis (0–4), as previously described [20]. Healthy controls had a normal Fibroscan® with a median < 7 kpa, a controlled attenuation parameter (CAP) value of < 255 dB/m, and normal values in all blood tests.

### Analyses of SCFAs

Venous blood samples were collected in EDTA tubes after at least four hours of fasting, immediately put on ice, and centrifuged within two hours of collection. Plasma was stored at − 80^0^C until analyses. The samples were analysed at Bevital AS, Bergen, Norway in 96-well plates with each plate including a fixed number of calibration and quality control samples. Using an isotope-labeled gas chromatography-tandem mass spectrometry (GC-MS/MS) platform with automated sample workup, we determined eight SCFAs (acetate, propionate, butyrate, formate, valerate, α-methylbutyrate, isovalerate, and isobutyrate) in plasma (https://bevital.no). Within- and between-day coefficient of variances for the eight SCFAs ranged from 3.3 to 9.3% and 2.3–5.9%, respectively. Additional information about quality control can be found at https://bevital.no/logistics/ and in supplementary information.

### Statistical analyses

Data are presented as n (%) or means ± standard deviations (SDs). All inferential tests are two-tailed with a nominal alpha level of 0.05. Adjustments for multiplicity were not performed due to the exploratory nature of the analyses. All statistical analyses were conducted with R v4.2.0 (https://www.r-project.org), and plots were made using the *ggplot2* v3.4.2 and *ggforrestplot* v0.1.0 packages.

In linear regression models of MASLD patients and healthy controls, we transformed SCFA concentrations by the natural logarithm and presented relative between-group differences as percentages calculated from the regression coefficients. In the figures, we show results in relative terms as sympercents (symmetric percent, s%), which are additive and symmetric percentage differences on the 100 log_e_ scale [21]. We log-transformed the SCFA concentrations by log2 in the logistic regression to show odds ratios with a doubling in SCFAs levels.

The associations between SCFA concentrations and MASLD (versus healthy controls) and histological MASLD severity (no/mild fibrosis versus significant fibrosis) were analysed with unmatched binomial logistic regression in age and sex adjusted analyses, as well as with adjustments for age, sex, and BMI. In the primary analysis, we used the *glm* function in the *stats* package v4.2.0 for the binominal coded outcome groups. To reduce possible bias introduced by small sample sizes, we repeated the analyses using penalized maximum likelihood logistic regression (Firth’s method) using the *logistf* function in the *logistf* package v1.24.1. The analyses largely confirmed our initial results and are reported in supplementary Tables 1 and 2.

Cross-sectional analyses of relative differences in SCFA concentrations between groups and fibrosis stages were performed by linear regression modeling using the *lm* function from the *stats* package v4.2.0 in models adjusted for (i) age and sex or (ii) age, sex, and BMI.

Left-censored missing values of SCFA concentrations due to lower than the limit of detection or quantification, were considered as missing rather than random, and imputed by the GSimp method, an approach previously utilized in metabolomics studies (see Supplementary method for further details) [22]. 

## Results

### Study participants

Patients with MASLD and healthy controls were matched for age and sex (Table 1). Patients with MASLD had higher HbA1c, ALT, and lipids than healthy controls. Fifty-one had type 2 diabetes, and 36 had dyslipidaemia. Histology showed that 51 had no/mild fibrosis (F0 *n* = 25, F1 *n* = 26) and 49 had significant fibrosis (F2 *n* = 20, F3 *n* = 12, F4 = 17). Severe steatosis was diagnosed in 66 patients (S2 *n* = 30, S3 *n* = 36) and lobular inflammation in 89 patients (grade 1 *n* = 62, grade 2 *n* = 22, grade 3 *n* = 2). Ballooning was identified in 80 MASLD patients (grade 1 *n* = 53, grade 2 *n* = 27).

**Table 1:** Characteristics of patients with MASLD and healthy controls

	Healthy controls (*n* = 50)	MASLD (*n* = 100)	*P*-value
Sex (male)	27 (54%)	58 (58%)	0.77
Age, years	50 (14)	51 (15)	0.51
BMI, kg/m^2^	24 (2.7)	35 (6.7)	< 0.001
HbA1c, mmol/mol	35 (3.7)	47 (13)	< 0.001
ALT, U/L	21 (6.5)	87 (79)	< 0.001
LDL-C, mmol/L	2.7 (0.86)	2.4 (0.98)	0.046
VLDL-C, mmol/L	0.42 (0.17)	1.0 (0.57)	< 0.001
HDL-C, mmol/L	1.8 (0.52)	1.1 (0.29)	< 0.001
Triglycerides, mmol/L	0.92 (0.39)	2.4 (1.5)	< 0.001
Fibroscan®, kpa	4.4 (1.2)	13 (8.4)	< 0.001
CAP, dB/m	210 (28)	340 (47)	< 0.001

*Data presented as n (%) or mean values with standard deviations. MASLD*, Metabolic dysfunction-associated steatotic liver disease; *BMI*, Body mass index; *ALT*, Alanine aminotransferase; *LDL-C*, Low-density lipoprotein cholesterol; *VLDL-C*, Very-low-density lipoprotein cholesterol; *HDL-C*, High-density lipoprotein cholesterol; *CAP*, Continuous attenuation factor

### Plasma SCFA levels in MASLD patients compared with healthy controls

**Fig. 1:**
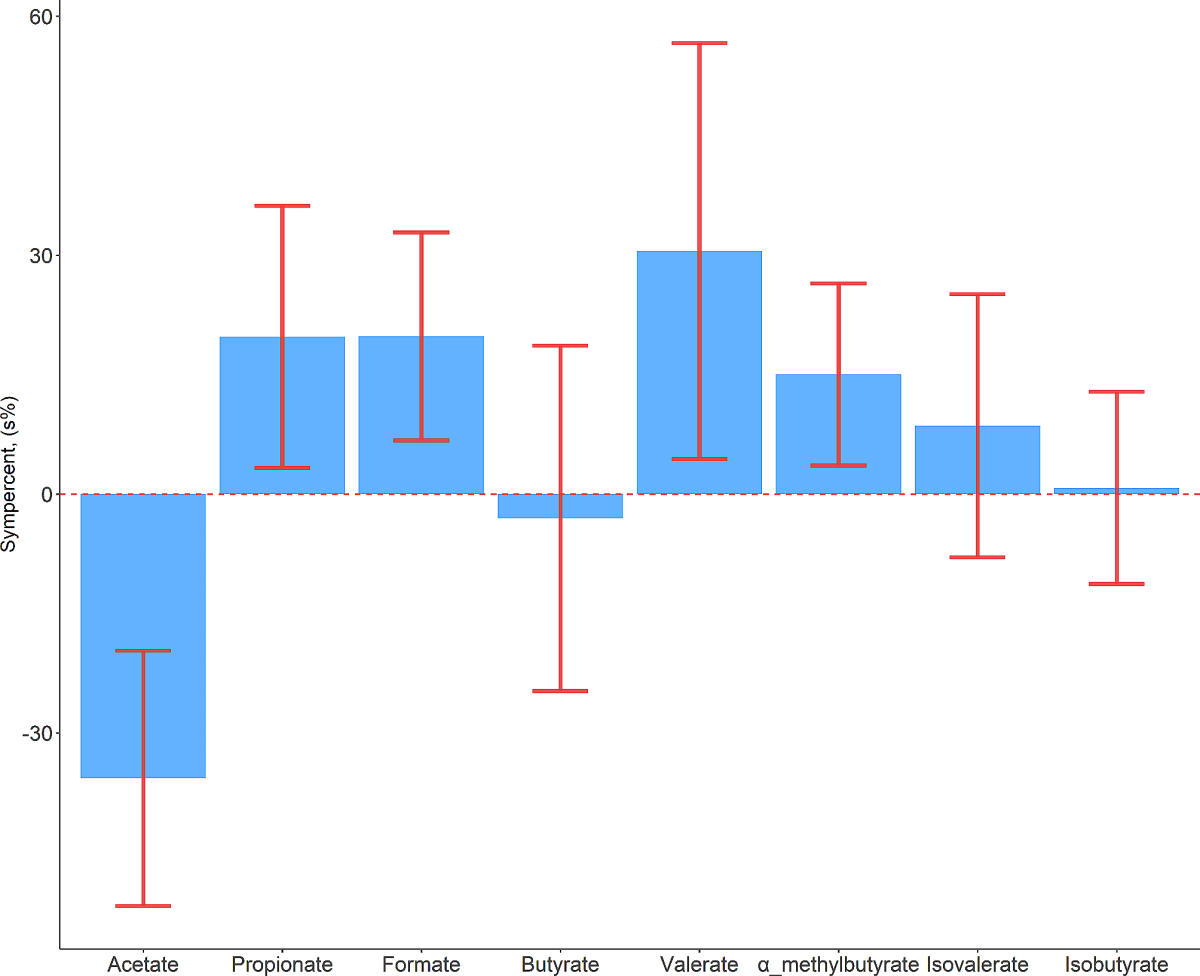
Percentage difference in SCFA concentrations between patients with MASLD and healthy controls expressed as sympercents (s%). Data were analysed by multivariable linear regression models adjusted for age and sex

In healthy controls, as well as in patients with MASLD, the SCFA with the highest concentration was acetate followed by formate and propionate (Table 2). The distributions of data points are shown by raincloud plots in Supplementary Fig. 1. Compared with healthy controls, patients with MASLD had significantly lower levels of acetate in age- and sex-adjusted analyses (− 30.0%, 95% CI − 40.4 to − 17.9, *p* < 0.001) and higher levels of propionate (21.8%, 95% CI 3.33 to 43.6, *p* = 0.02), formate (21.9%, 95% CI 6.99 to 38.9, *p* = 0.003), valerate (35.7%, 95% CI 4.53 to 76.2, *p* = 0.02), and α-methylbutyrate (16.2%; 95% CI 3.66 to 30.3, *p* = 0.01), but not butyrate, isobutyrate, or isovalerate (Fig. 1). When additionally adjusting for BMI, the difference was no longer statistically significant for acetate and valerate (Supplementary Table 3).

**Table 2:** Concentration of plasma SCFAs in healthy controls and patients with MASLD

SCFA, µmol/L	Healthy controls *N* = 50	MASLD *N* = 100
Acetate	57.6 (*25.6*)	44.8 (*65.1*)
Propionate	1.25 (*0.54)*	1.71 (*1.48)*
Butyrate	0.68 (*0.50)*	0.71 (*0.99)*
Formate	18.9 (*6.96)*	23.9 (*11.1)*
Valerate	0.063 (*0.044)*	0.11 (*0.18)*
α-methylbutyrate	0.15 (*0.040)*	0.19 (*0.11)*
Isovalerate	0.50 (*0.26)*	0.55 (*0.25)*
Isobutyrate	0.29 (*0.065)*	0.32 (*0.23)*

**Fig. 2:**
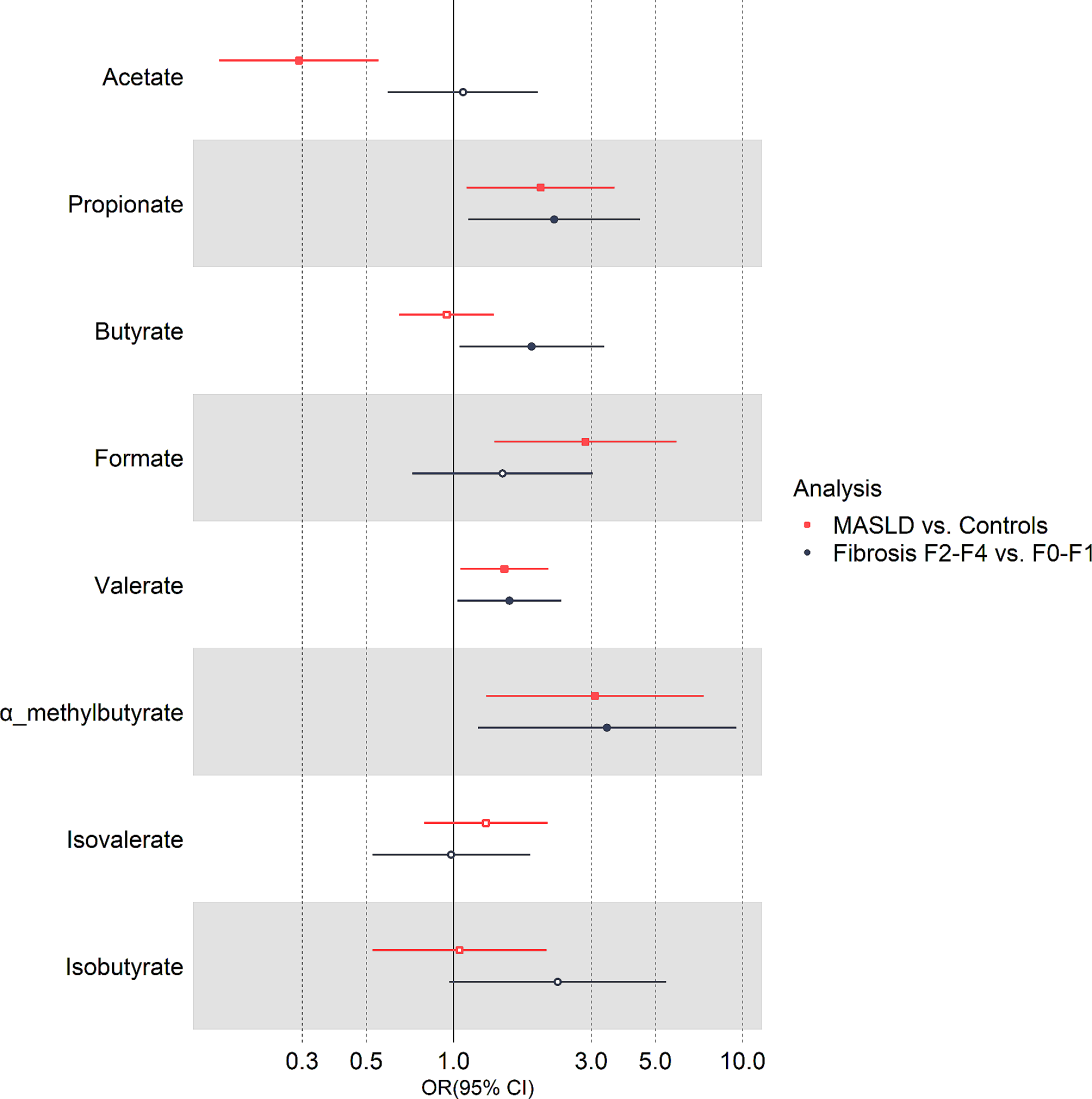
Adjusted OR from logistic regression analysis evaluating healthy controls versus patients with MASLD (black lines) and patients with MASLD and no/mild fibrosis versus significant fibrosis (red lines). Analyses are adjusted for age and sex

In the logistic regression analyses adjusted for age and sex (Fig. 2), the odds of having MASLD was inversely associated with a doubling of the plasma concentration of acetate (adjusted odds ratio (OR) = 0.29, 95% CI 0.16 to 0.55, *p* < 0.001), while a positive relationship was found for propionate (OR = 2.00, 95% CI 1.11 to 3.61, *p* = 0.02), formate (OR = 2.86, 95% CI 1.39 to 5.91, *p* = 0.004), valerate (OR = 1.50, 95% CI 1.06 to 2.13, *p* = 0.02), and α-methylbutyrate (OR = 3.09, 95% CI 1.30 to 7.34, *p* = 0.01). No significant associations were found for butyrate, isobutyrate, or isovalerate (Fig. 2). When additionally controlling for BMI, the association was no longer statistically significant for acetate and valerate (Supplementary Table 4).

### Plasma SCFA levels in MASLD according to histological severity

Logistic regression analyses adjusted for age and sex found a positive association between significant fibrosis and plasma propionate (OR 2.23; 95% CI 1.13 to 4.43, *p* = 0.02), butyrate (OR 1.87; 95% CI 1.50 to 3.32, *p* = 0.03), valerate (OR 1.56; 95% CI 1.03 to 2.36, *p* = 0.03), and α-methylbutyrate (OR 3.40; 95% CI 1.22 to 9.5, *p* = 0.02) concentrations (Fig. 2; Table 3). The results remained significant after additional adjustment for BMI.

**Table 3:** Logistic regression analysis evaluating SCFAs in patients with MASLD grouped according to histological severity

SCFAs	Fibrosis OR (95% CI) *p*	Steatosis OR (95% CI) *p*	Lobular inflammation OR (95% CI) *p*	Ballooning OR (95% CI) *p*
Acetate	1.08 (0.59–1.96) 0.80	0.74 (0.4–1.38) 0.35	0.87 (0.38–1.99) 0.74	0.86 (0.44–1.68) 0.65
Propionate	2.23 (1.13–4.43) *0.02*	*0.38* *(0.19–0.74)* *0.004*	*0.61* *(0.29–1.28)* *0.19*	*0.94* *(0.48–1.82)* *0.85*
Formate	1.48 (0.72–3.04) 0.29	0.57 (0.27–1.20) 0.14	0.50 (0.17–1.46) 0.21	1.06 (0.46–2.47) 0.89
Butyrate	1.87 (1.50–3.32) *0.03*	*0.75* *(0.46–1.22)* *0.25*	*0.71* *(0.37–1.33)* *0.28*	*0.92* *(0.53–1.60)* *0.76*
Valerate	1.56 (1.03–2.36) *0.03*	*0.78* *(0.54–1.12)* *0.18*	*0.81* *(0.49–1.35)* *0.42*	*1.13* *(0.72–1.77)* *0.59*
α-methylbutyrate	3.40 (1.22–9.5) *0.02*	*0.34* *(0.14–0.86)* *0.02*	*0.31* *(0.10–0.93)* *0.04*	*0.58* *(0.23–1.45)* *0.25*
Isobutyrate	2.30 (0.97–5.44) 0.06	*0.33* *(0.014–0.80)* *0.01*	*0.38* *(0.14–1.02)* *0.054*	*0.75* *(0.34–1.73)* *0.52*
Isovalerate	0.98 (0.52–1.84) 0.96	0.59 (0.29–1.22) 0.15	0.57 (0.19–1.74) 0.33	1.06 (0.53–2.15) 0.86

Adjusted OR with (95% CI) and *p* values from logistic regression analysis evaluating SCFAs in patients with MASLD grouped according to histological severity. The analyses evaluate fibrosis (significant, F2-F4), steatosis (severe, S2/3) and the presence of lobular inflammation and ballooning. Analyses are adjusted for age and sex. *SCFAs* Short chain fatty acids

In the age- and sex-adjusted logistic regression analyses, severe steatosis (S2/3) was inversely associated with plasma propionate (OR 0.38; 95% CI 0.19 to 0.74, *p* = 0.004), α-methylbutyrate (OR 0.34; 95% CI 0.14 to 0.86, *p* = 0.02), and iso-butyrate (OR 0.33; 95% CI 0.14 to 0.80, *p* = 0.01) concentrations. The only significant association for the presence of lobular inflammation was α-methylbutyrate (OR 0.31; 95% CI 0.10 to 0.93, *p* = 0.04), and no associations between SCFAs and ballooning were identified (Table 3).

**Fig. 3:**
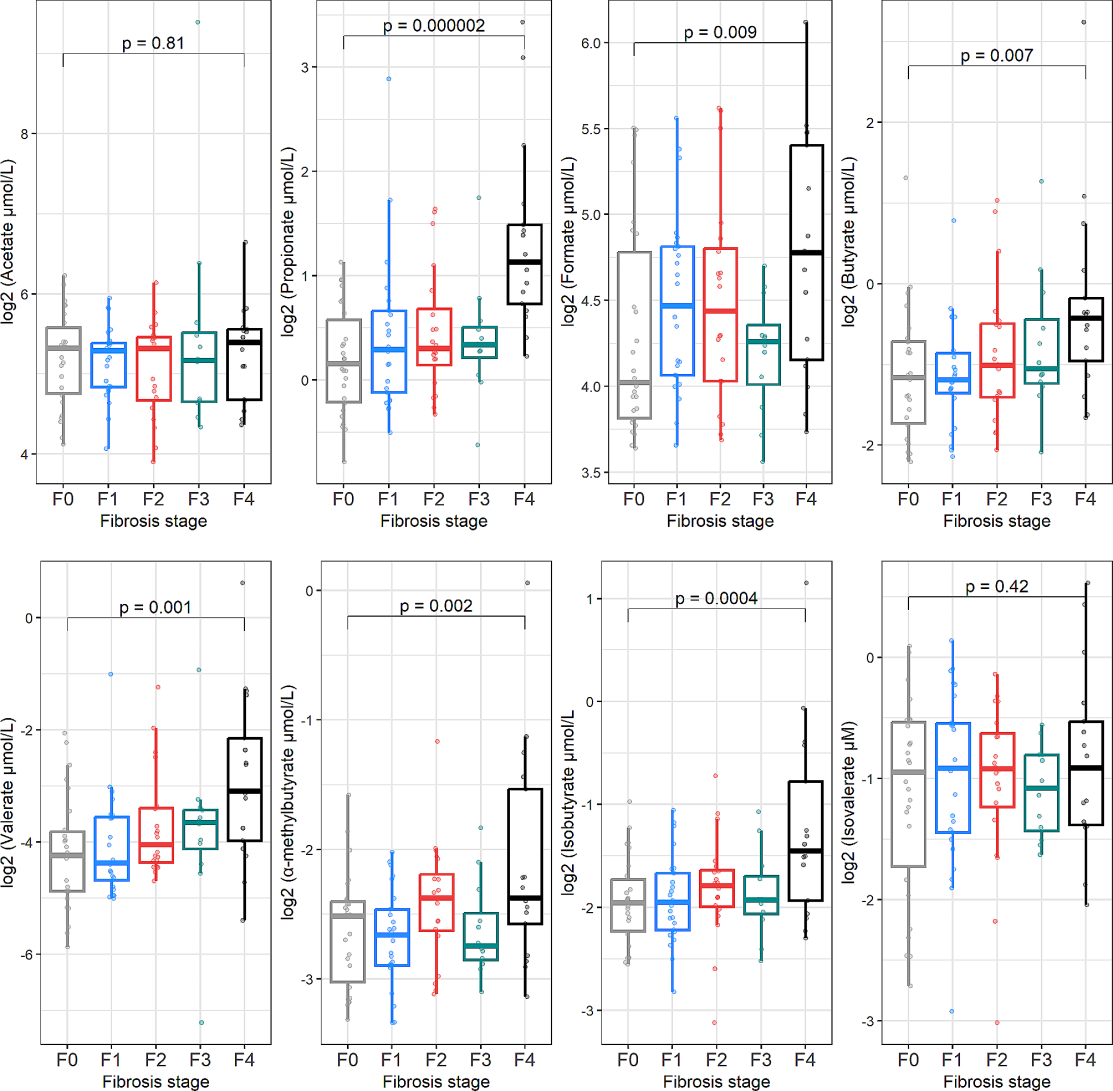
SCFAs concentrations for each fibrosis group. Data presented as boxplots of median log2-transformed SCFAs concentrations for each fibrosis group (F0 *n* = 26, F1 *n* = 25, F2 *n* = 20, F3 *n* = 12, F4 *n* = 17). *P*-values from linear regression models adjusted for age and sex (Supplementary Table 5)

When exploring plasma concentrations of SCFAs according to different fibrosis stages (Fig. 3, Supplementary Table 5), we found no significant differences for acetate and isovalerate in the linear regression modeling adjusting for age and sex., Among the remaining SCFAs, all had increased plasma concentrations in patients with MASLD cirrhosis. Compared to the group of MASLD patients with F0 fibrosis, F4 fibrosis patients had higher plasma concentrations of propionate (115%, 95% CI 59.3 to 190, *p* < 0.001), formate (41.7%; 95% CI 9.14 to 84.0, *p* = 0.009), butyrate (70.7%; 95% CI 16.4 to 150, *p* = 0.007), valerate (130%; 95% CI 39.1 to 279, *p* = 0.001), α-methylbutyrate (41.4%; 95% CI 13.4 to 76.3, *p* = 0.002), and isobutyrate (57.1%; 95% CI 23.1 to 100, *p* < 0.001).

## Discussion

We found that the odds of having MASLD was associated with lower plasma concentrations of acetate and higher concentrations of propionate, formate, valerate, and α-methylbutyrate. The concentration of acetate was not associated with the histological severity of MASLD based on fibrosis severity (comparing severe fibrosis versus no/mild fibrosis), but we found that significant fibrosis was associated with increased propionate, butyrate, valerate, and α-methylbutyrate concentrations.

Acetate and formate had the highest plasma concentrations in our study, with plasma concentrations more than ten times higher than the third most abundant SCFA, propionate. The high levels of formate arise from both endogenous production and production from the gut microbes [23]. Acetate, propionate, and butyrate are the most abundant SCFAs in the gut, produced from saccharolytic fermentation of dietary fibers, in contrast to the less abundant SCFAs from proteolytic fermentation. In general, saccharolytic SCFAs are thought to have beneficial systemic effects on glucose and lipid metabolism, as well as on the regulation of satiety and inflammation, whereas proteolytic SCFAs are less well studied but often thought to have harmful systemic effects [24]. 

In our study, both acetate and propionate were associated with MASLD. While the exact role of these SCFAs in MASLD is unknown, indirect evidence may be derived via studies evaluating other metabolic diseases. Acetate has previously been linked with gut microbiota diversity, lower visceral fat, and milder cases of MASLD [25, 26]. In agreement with these previous findings, patients with MASLD had a lower acetate concentration compared with healthy controls in our study. Propionate is also positively associated with health in adequate concentrations and has been linked with the release of gut hormones affecting energy intake and satiety [27]. However, studies indicating negative effects also exist. In a study of patients with early MASLD, increased abundance of SCFAs-producing bacteria and fecal acetate and propionate levels were associated with a higher TH17/rTreg ratio, suggesting that SFCAs could contribute to low-grade inflammation [6]. Increased fecal propionate has been associated with increased risk of type 2 diabetes [28], and supplementation with propionate has been found to increase plasma levels of glucagon and insulin, increasing the risk of insulin resistance and weight gain [14, 15]. In agreement with these findings, our study found higher plasma concentrations of propionate in patients with MASLD, who also had higher HbA1c, BMI, and prevalence of diabetes.

MASLD is characterized by specific histological changes in the liver, including steatosis, inflammation, ballooning, and fibrosis. We evaluated the plasma SCFAs in relation to histological features in patients with MASLD evenly distributed across the five fibrosis categories, representing the entire spectrum from simple steatosis to metabolic dysfunction-associated steatohepatitis and cirrhosis. In a previous study investigating the gut microbiome in MASLD patients, host enzymes associated with propionate and butyrate metabolism were more abundant in advanced fibrosis than in mild/moderate fibrosis [26]. In the present study, we found higher concentrations of both propionate and butyrate in patients with significant fibrosis compared to patients with MASLD and no/mild fibrosis. Behary et al. found increased serum levels of both propionate and butyrate in patients with MASLD-cirrhosis and hepatocellular carcinoma and ex vivo studies, suggesting potential immune-modulatory effects [17]. However, Xiong et al. found that plasma concentrations of propionate and butyrate were decreased in MASLD-cirrhosis compared with patients classified as having MASLD without fibrosis based on clinical assessments [13]. The contrasting findings may be due to the small sample sizes and heterogeneity of the studied population, underscoring the need for larger, clinical studies including a broad spectrum of MASLD patients.

Previous studies investigating circulating SCFAs in relation to MASLD present inconsistent findings which may reflect a lack of standardization and differences in the study design [13, 16–18]. The selection of both patients and controls makes it difficult to compare results across studies. For instance, two studies included patients with MASLD cirrhosis diagnosed clinically or histologically, and one study included controls with increased BMI as well as other metabolic diseases [13, 17]., while another study only included participants with MASLD without fibrosis and controls undergoing gastric bypass surgery [16]. 

In a study including participants with steatotic liver disease and type 2 diabetes, Tsai et al. found that those with the greatest degree of steatosis (assessed by ultrasound) tended to have similar circulating concentrations of most SCFAs as those with ”no/mild steatosis”, however, isobutyrate, and methylbutyrate levels were lower in participants with “moderate/severe steatosis” [18]. We found a negative association between severe histological steatosis (S2-3) and propionate, α-methylbutyrate, and isobutyrate. Our observations may reflect alterations in lipid metabolism, potentially linked to gut dysbiosis and the gut-liver axis. However, a study including participants undergoing bariatric surgery found no differences in circulating SCFA concentrations between participants with normal liver tissue, simple steatosis, or MASLD without fibrosis [16]. 

The concentrations of propionate and butyrate but not acetate may be higher in the portal vein compared to the hepatic vein, indicating uptake of these SCFAs in the liver [8]. We found higher SCFA concentrations in patients with MASLD-cirrhosis, which may reflect portosystemic shunts or the impaired function of the cirrhotic liver decreasing SCFA uptake and metabolism by the liver. Clausen et al. found higher SCFA concentrations in patients with hepatic coma compared to both patients with cirrhosis and healthy controls. In contrast, Bloemen et al. found preserved butyrate and propionate liver uptake in 12 cirrhotic patients, and Juanola et al. found an inverse relationship between circulating SCFAs and hepatic venous-pressure gradient (HVPG) measure in cirrhotic patients, though only reaching significance for butyrate [29–31]. In these studies, the etiology of cirrhosis was primarily alcohol, which could also affect SCFA concentrations through reduced intake of dietary fiber, and the health and diversity of the patients gut microbiome may differ from that found in MASLD.

In our study, blood samples were collected after a four hour fasting period. The duration of fasting could potentially influence the concentration of SCFAs in the blood as this will affect the metabolic processes in the body. Fasting can also lead to alterations in the gut microbiota and their production of SCFAs the availability of substrates for bacterial fermentation in the gut. Therefore, fasting can impact SCFA concentrations in the blood. The duration of fasting, dietary differences, use of probiotics, and medication could potentially affect our results including differences between patients with MASLD and controls [32–34]. There is no clear evidence showing the effect of diet or medication on SCFAs but a systematic review found no clear effect of supplementary dietary fibers on SCFA levels [34]. In addition, a review evaluating the effects of fasting found a possible benefit of fasting on gut microbiota but that additional human models are needed [32]. It is possible that dietary modifications can change the composition as well as the diversity of the microbiota and subsequently the progression of MASLD. Additional studies are needed to identify the diets that could lead to clinically significant beneficial changes and the clinical value of these.Likewise, different medications are likely to be important in the assessment of gut microbiota. For example, although the exact mechanisms are not yet fully elucidated, the use of statins might alter the lipid profile and systemic inflammation and eventually influence the gut environment and microbiota. Proton pump inhibitors could also impact the balance of bacteria in the upper gastrointestinal tract due to their effect on reduced acidity in the stomach. The use of laxatives, antibiotics, probiotics and other medications are also likely to influence microbiota. Due to the observational nature, we were unable to control for these potential confounders. The included patients received several different medications and detailed evaluations into the impact of each drug are not possible. However, future studies evaluating possible detrimental as well as beneficial effects would provide important information. One possibly beneficial medication is prebiotics as well as probiotics. Both could potentially modulate the composition and diversity of the gut microbiota and/or restore a healthier balance of gut microbes. However, more research is needed to better understand the specific strains, dosages, and mechanisms by which both exert their effects in MASLD.

## Conclusions

In the present study, lower plasma concentrations of acetate were associated with having MASLD, whereas higher concentrations of propionate, valerate, and α-methylbutyrate were associated with both MASLD and significant fibrosis. Our findings could indicate a role for SCFAs in MASLD and disease progression. However, previous results are somewhat contradicting, and differences in patients and study design make it difficult to compare across studies. To gain more knowledge on the potential role of SCFAs in MASLD and cirrhosis, validation studies, greater standardization, and larger clinical studies including a broad spectrum of MASLD patients are needed.

### Electronic supplementary material

Below is the link to the electronic supplementary material.

**Supplementary Table 1** Associations between SCFA concentrations and MASLD status, sensitivity analysis using penalized maximum likelihood logistic regression (Firth’s method). **Supplementary Table 2** Odds of having significant fibrosis (F2?F4 vs. F0?F1), steatosis (grade 2?3 vs. grade 0?1), presence of lobular inflammation (grade 1?3 vs. grade 0), and presence of ballooning (grade 1?2 vs. grade 0) (sensitivity analysis using Firth’s method). **Supplementary Table 3** Relative differences in SCFA levels between groups (MASLD vs. HC). **Supplementary Table 4** Associations between SCFA concentrations and MASLD status, analysed using logistic regression models. **Supplementary Table 5** Relative between-fibrosis stage differences in SCFA levels (F0 as the reference group). **Supplementary Fig. 1** Raincloud plots showing the distribution of log2-transformed raw data points for the eight measured SCFAs

## Data Availability

The datasets generated and analysed during the current study are not publicly available due to Danish Legislation, where sharing of individual patient related data is not permitted without thorough anonymization. Selected anonymized data for main findings are available from the corresponding author on reasonable request.

## Abbreviations

MASLD: Metabolic dysfunction associated steatotic liver disease
SCFAs: Short chain fatty acids
CAP: Controlled attenuation parameter
GC-MS/MS: Gas chromatography-tandem mass spectrometry
BMI: Body mass index
ALT: Alanine aminotransferase
LDL-C: Low-density lipoprotein cholesterol
VLDL-C: Very-low-density lipoprotein cholesterol
HDL-C: High-density lipoprotein cholesterol
