# Supplementary material for: Targeted metabolomics reveals plasma short-chain fatty acids are associated with metabolic dysfunction-associated steatotic liver disease
Source: BMC Gastroenterol. 2024 Jan 23;24:43. doi: 10.1186/s12876-024-03129-7 (PMC10804800; doi:10.1186/s12876-024-03129-7)
Supplement: Supplementary file 1 — Supplementary Table 1 Associations between SCFA concentrations and MASLD status, sensitivity analysis using penalized maximum likelihood logistic regression (Firth’s method). Supplementary Table 2 Odds of having significant fibrosis (F2?F4 vs. F0?F1), steatosis (grade 2?3 vs. grade 0?1), presence of lobular inflammation (grade 1?3 vs. grade 0), and presence of ballooning (grade 1?2 vs. grade 0) (sensitivity analysis using Firth’s method). Supplementary Table 3 Relative differences in SCFA levels between groups (MASLD vs. HC). Supplementary Table 4 Associations between SCFA concentrations and MASLD status, analysed using logistic regression models. Supplementary Table 5 Relative between-fibrosis stage differences in SCFA levels (F0 as the reference group). Supplementary Fig. 1 Raincloud plots showing the distribution of log2-transformed raw data points for the eight measured SCFAs [file 12876_2024_3129_MOESM1_ESM.docx]

**Supplementary material:**

| Supplementary Table 1 Associations between SCFA concentrations and MASLD status, sensitivity analysis using penalized maximum likelihood logistic regression (Firth’s method). | | | |
| --- | --- | --- | --- |
| SCFAs | Model 1 | Model 2 | Model 3 |
| Acetate | 0.30  (0.16 – 0.56)  0.00002 | 0.31  (0.17 – 0.57)  0.00002 | 0.59  (0.32 – 1.11)  0.12 |
| Proprionate | 1.98  (1.13 – 3.48)  0.01 | 1.92  (1.09 – 3.40)  0.02 | 2.31  (1.07 – 4.50)  0.03 |
| Formate | 2.75  (1.35 – 5.61)  0.003 | 2.72  (1.35 – 5.48)  0.003 | 3.83  (1.43 – 10.25)  0.005 |
| Butyrate | 0.98  (0.68 – 1.40)  0.90 | 0.94  (0.65 – 1.36)  0.76 | 0.92  (0.55 – 1.55)  0.77 |
| Valerate | 1.49  (1.07 – 2.09)  0.01 | 1.47  (1.05 – 2.06)  0.02 | 1.21  (0.72 – 2.03)  0.47 |
| α-methylbutyrate | 2.96  (1.30 – 6.73)  0.006 | 2.91  (1.26 – 6.71)  0.008 | 5.23  (1.38 – 19.84)  0.003 |
| Isobutyrate | 1.10  (0.58 – 2.10)  0.77 | 1.03  (0.53 – 1.99)  0.93 | 2.11  (0.86 – 5.2)  0.11 |
| Isovalerate | 1.34  (0.84 – 2.11)  0.21 | 1.29  (0.80 – 2.08)  0.31 | 1.76  (0.83 – 3.73)  0.16 |

Model 1 unadjusted. Model 2 adjusted for age and sex. Model 3 adjusted for age, sex, and BMI. Presented as OR, (95%CI) and p-values. SCFAs = short-chain fatty acids

| Supplementary Table 2 Odds of having significant fibrosis (F2–F4 vs. F0–F1), steatosis (grade 2–3 vs. grade 0–1), presence of lobular inflammation (grade 1–3 vs. grade 0), and presence of ballooning (grade 1–2 vs. grade 0) (sensitivity analysis using Firth’s method). | | | | |
| --- | --- | --- | --- | --- |
| SCFAs | Significant fibrosis  (F2–F4) | Significant steatosis  (S2–S4) | Lobular inflammation  present | Ballooning  present |
| Acetate | 0.92  (0.54 – 1.55)  0.75 | 0.58  (0.32 -1.06)  0.06 | 0.60  (0.33 – 1.08)  0.08 | 0.66  (0.38 – 1.15)  0.13 |
| Propionate | 2.21  (1.18 – 4.15)  0.007 | *0.53*  *(0.30 – 0.92)*  *0.02* | *0.90*  *(0.50 – 1.63)*  *0.74* | *1.12*  *(0.63 – 1.99)*  *0.70* |
| Formate | 1.72  (0.87 – 3.41)  0.11 | 0.86  (0.46 – 1.63)  0.65 | 1.19  (0.55 – 2.55)  0.66 | 1.52  (0.74 – 3.13)  0.25 |
| Butyrate | 1.56  (0.92 – 2.52)  0.06 | *0.75*  *(0.49 – 1.15)*  *0.19* | *0.73*  *(0.46 – 1.17)*  *0.20* | *0.86*  *(0.55-1.34)*  *0.76* |
| Valerate | 1.60  (1.08 – 2.38)  0.01 | *0.88*  *(0.63 – 1.22)*  *0.43* | *1.00*  *(0.67 – 1.49)*  *1.00* | *1.23*  *(0.83 – 1.83)*  *0.29* |
| α-methylbutyrate | 3.31  (1.26 – 8.69)  0.008 | *0.49*  *(0.22 – 1.10)*  *0.072* | *0.59*  *(0.26 – 1.38)*  *0.24* | *0.77*  *(0.35 – 1.70)*  *0.53* |
| Isobutyrate | 2.01  (0.92 – 4.40)  0.07 | *0.35*  *(0.15 – 0.79)*  *0.006* | *0.47*  *(0.22 – 1.02)*  *0.06* | *0.73*  *(0.36 – 1.49)*  *0.39* |
| Isovalerate | 1.05  (0.59 – 1.87)  0.87 | 0.80  (0.46 – 1.40)  0.43 | 1.03  (0.55 – 1.94)  0.92 | 1.22  (0.69 – 2.16)  0.49 |

Presented as OR, (95%CI) and p-values.

| Supplementary Table 3 Relative differences in SCFA levels between groups (MASLD vs. HC). | | | |
| --- | --- | --- | --- |
| SCFAs | Model 1 | Model 2 | Model 3 |
| Acetate | -29.89 %  (-40.19 - -17.83)  0.00002 | -30.01 %  (-40.38 - -17.85)  *0.00002* | -10.34%  (-27.39 - 10.73)  *p = 0.31* |
| Proprionate | *23.92%*  *(4.83 - 46.47)*  *0.01* | 21.82 %  (3.33 - 43.63)  *0.02* | 32.66 %  (6.00 - 66.03)  *p = 0.01* |
| Formate | *21.67%*  *(6.91 - 38.47)*  *0.003* | 21.91 %  (6.99 – 38.90)  *0.003* | 45.45 %  (22.29 – 73.00)  *p = 0.00004* |
| Butyrate | -1.15%  (-20.56 – 23.00)  0.92 | -2.99 %  (-21.89 - 20.49)  *0.8* | 4.09 %  (-22.58 - 39.97)  *p = 0.8* |
| Valerate | *39.53%*  *(6.98 - 81.99)*  *0.01* | 35.71 %  (4.53 - 76.18)  *0.02* | 28.72 %  (-9.92 - 83.93)  *p = 0.16* |
| α-methylbutyrate | *17.53%*  *(4.58 - 32.08)*  *0.007* | 16.22 %  (3.66 - 30.29)  *0.01* | 22.98 %  (5.24- 43.72)  *p = 0.01* |
| Isobutyrate | *2.16*  *(-9.67 -* *15.55)*  *0.73* | 0.8 %  (-10.66 - 13.72)  *0.90* | 3.90 %  (-11.91 - 22.53)  *p = 0.65* |
| Isovalerate | *11.53%*  *(-6.17 - 32.59)*  *0.21* | 8.97 %  (-7.61 - 28.53)  *0.31* | 21.63 %  (-2.81 - 52.23)  *p = 0.87* |

Linear regression models. Model 1 unadjusted. Model 2 adjusted for age and sex. Model 3 adjusted for age, sex, and BMI. Percentages calculated from the regression coefficients (100 x (exp^estimate^ – 1)). Presented as estimate, (95%CI) and p-values.

SCFAs = short-chain fatty acids

| Supplementary Table 4 Associations between SCFA concentrations and MASLD status, analysed using logistic regression models | | | |
| --- | --- | --- | --- |
| SCFAs | Model 1 | Model 2 | Model 3 |
| Acetate | 0.29  (0.16 – 0.55)  0.0001 | 0.29  (0.15 – 0.55)  0.0001 | 0.57  (0.28 – 1.16)  0.12 |
| Propionate | 2.05  (1.15 – 3.64)  0.01 | 2.00  (1.11 – 3.61)  0.02 | 2.54  (1.08 – 5.97)  0.03 |
| Formate | 2.86  (1.38 – 5.91)  0.005 | 2.86  (1.39 – 5.91)  0.004 | 4.37  (1.49 – 12.86)  0.007 |
| Butyrate | 0.98  (0.68 – 1.42)  0.92 | 0.95  (0.65 – 1.38)  0.78 | 0.92  (0.52 – 1.63)  0.78 |
| Valerate | 1.52  (1.08 – 2.14)  0.02 | 1.50  (1.06 – 2.13)  0.02 | 1.26  (0.71 – 2.22)  0.43 |
| α-methylbutyrate | 3.09  (1.33 – 7.19)  0.009 | 3.09  (1.30 – 7.34)  0.01 | 7.12  (1.45 – 34.93)  0.02 |
| Isobutyrate | 1.13  (0.58 – 2.20)  0.73 | 1.05  (0.53 – 2.10)  0.89 | 2.63  (0.81 – 6.85)  0.11 |
| Isovalerate | 1.34  (0.84 – 2.14)  0.21 | 1.29  (0.79 – 2.12)  0.30 | 1.84  (0.81 – 4.20)  0.15 |

Model 1 unadjusted. Model 2 adjusted for age and sex. Model 3 adjusted for age, sex, and BMI. Presented as OR (95%CI) and p-values. SCFAs = short chain fatty acids

| Supplementary Table 5 Relative between-fibrosis stage differences in SCFA levels (F0 as the reference group). | | | | |
| --- | --- | --- | --- | --- |
| SCFAs | F1 Fibrosis | F2 Fibrosis | F3 Fibrosis | F4 Fibrosis |
| Acetate | -1.55%  (-25.19 – 29.56)  0.91 | -8.41%  (-31.73 – 22.87)  0.55 | 19.59  (-15.23 – 68.72)  0.30 | 3.97  (-24.31 – 42.82)  0.81 |
| Propionate | *18.33%*  *(-8.63 – 53.24)*  *0.20* | *19.43%*  *(-9.43 – 57.50)*  *0.21* | *16.30%*  *(-15.89 – 60.82)*  *0.36* | *114.83%*  *(59.32 – 189.70)*  *0.000002* |
| Formate | 13.51%  (-9.45 – 42.28)  0.27 | 16.55%  (-8.48 – 48.42)  0.21 | -6.30%  (-29.40 – 24.38)  0.65 | 41.72%  (9.14 – 84.03)  0.009 |
| Butyrate | *5.20%*  *(-24.46 - 46.52)*  *0.76* | *18.15%*  *(-17.11 - 68.41)*  *0.35* | *34.12%*  *(-11.45 - 103.15)*  *0.16* | *70.70%*  *(16.38 - 150.38)*  *0.007* |
| Valerate | *16.34%*  *(-24.61 - 79.54)*  *0.50* | *34.97%*  *(-15.16 - 114.70)*  *0.20* | *30.53%*  *(-24.22 - 124.84)*  *0.33* | *129.67%*  *(39.07 - 279.31)*  *0.001* |
| α-methylbutyrate | *-1.42%*  *(-18.55 - 19.31)*  *0.88* | *16.21%*  *(-5.26 - 42.54)*  *0.15* | *4.54%*  *(-17.70 - 32.80)*  *0.71* | *41.39%*  *(13.39 - 76.31)*  *0.002* |
| Isobutyrate | 4.25%  (-15.57 - 28.72)  0.70 | *8.12%*  *(-13.72 - 35.49)*  *0.49* | *6.97%*  *(-17.87 - 39.33)*  *0.61* | *57.07%*  *(23.09 - 100.44)*  *0.0004* |
| Isovalerate | 16.51%  (-11.17 - 52.81)  0.27 | 4.40%  (-21.90 – 39.56)  0.77 | 4.80%  (-25.40 - 47.22)  0.78 | 13.51%  (-17.05 - 55.32)  0.42 |

Multiple linear regression model adjusting for age and sex. Percentages calculated from the regression coefficients (100 x (exp^estimate^ – 1)). Presented as estimates, (95%CI) and p-values.

*SCFA* Short-chain fatty acids.


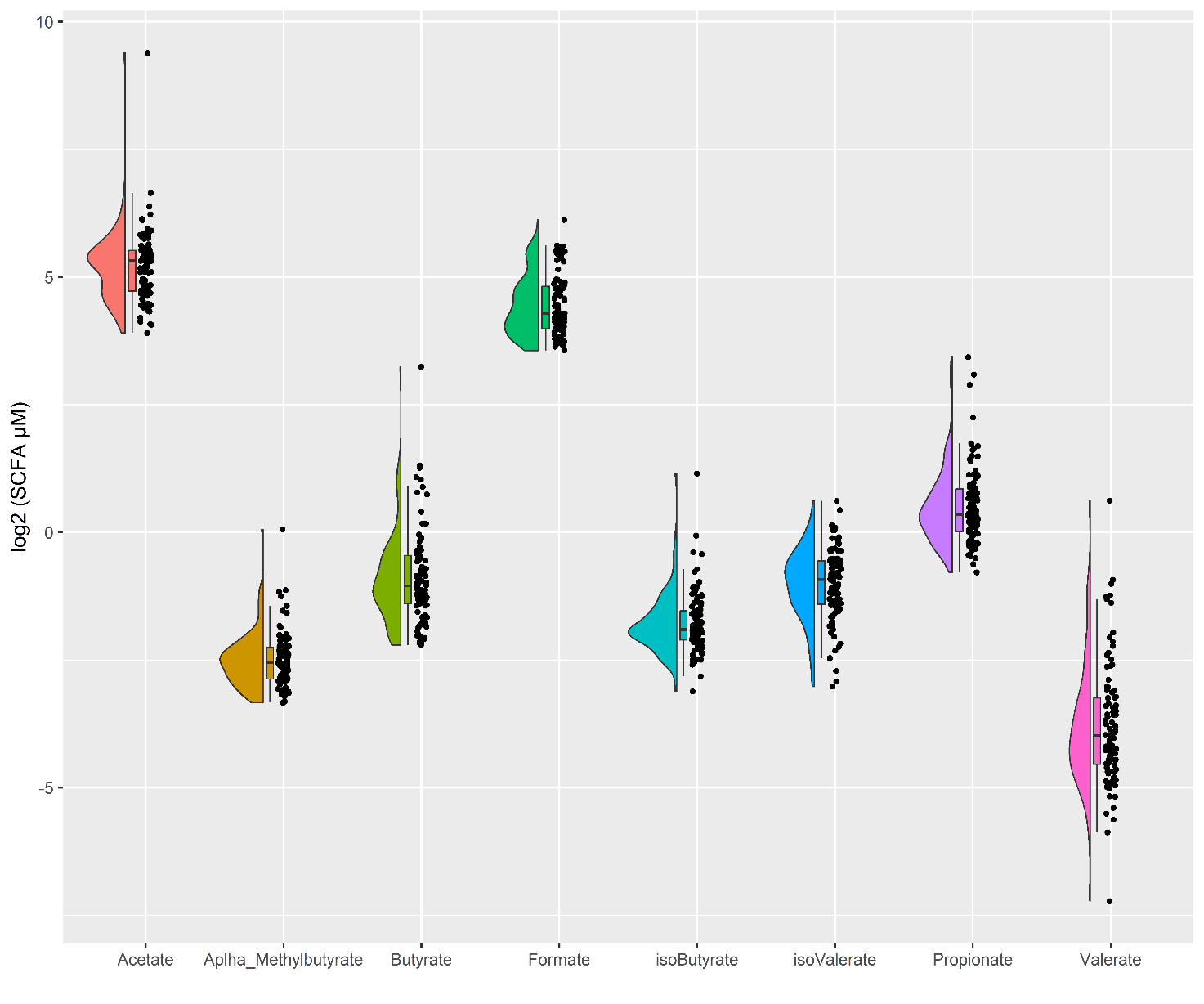


**Supplementary Fig. 1** Raincloud plots showing the distribution of log2-transformed raw data points for the eight measured SCFAs

**Supplementary Method:**

**Imputation of left-censored missing values**

GSimp is a Gibbs sampler-based left-censored missing value imputation procedure that utilizes the predictive information of other variables by employing a prediction model and simultaneously held a truncated normal distribution for each missing element. Missing values were initialized by quantile regression imputation of left-censored data (QRILC). Data was natural log-transformed before QRILC was conducted to improve the imputation accuracy and ensure positive values in the original scale after back-transformation. Elastic net from the R package *glmnet* was used as the prediction model. The minimum observed value of the missing variable was applied as an informative upper truncation point and -Inf as a non-informative lower truncation point for left-censored missing (1).

1. Wei R, Wang J, Jia E, Chen T, Ni Y, Jia W. GSimp: A Gibbs sampler based left-censored missing value imputation approach for metabolomics studies. PLoS Comput Biol. 2018;14(1):1–14.

**Quality Assessment Measures**

The analyses were performed at Bevital, Norway. As described at <https://bevital.no/logistics/>, each set of 96 vials contains 6 vials with calibrators, 3 with control plasma samples with known biomarker concentrations and one vial without biomarker (blank, to control for carry over). The calibrators are diagonally located (from upper left to lover right corner) across the sample tray to verify positioning of the tray in the autosampler. Large stock solutions of plasma calibrator and control plasma are prepared in sufficient amount to last for years, to minimize chance of assay drift over time. These stocks are aliquoted and stored at -80 °C. New stock are calibrated by comparison with the previous validated stock solution by analysing about 1000 parallel samples over one month.
